# Oral cancer cell to endothelial cell communication via exosomal miR-21/RMND5A pathway

**DOI:** 10.1186/s12903-024-03852-3

**Published:** 2024-01-16

**Authors:** Yu-qi Sun, Bing Wang, Lin-wei Zheng, Ji-hong Zhao, Jian-gang Ren

**Affiliations:** 1 The State Key Laboratory Breeding Base of Basic Science of Stomatology (Hubei-MOST) & Key Laboratory of Oral Biomedicine Ministry of Education, School & Hospital of Stomatology, Wuhan University, No. 237 Luoyu Road, Wuhan, 430079 China; 2 Department of Oral and Maxillofacial Surgery, School & Hospital of Stomatology, Wuhan University, Wuhan, 430079 China

**Keywords:** Required for meiotic nuclear division 5 homolog A, Oral cancer, Endothelial cells, Exosome, miR-21

## Abstract

Required for meiotic nuclear division 5 homolog A (RMND5A), a novel ubiquitin E3 Ligase, has been reported to correlate with poor prognosis of several cancers. However, its role in endothelial cells has not been reported. In this study, overexpression of RMND5A in human umbilical vein endothelial cells (HUVECs) was performed via lentiviral infection, followed by MTT, would healing and tube formation assay as well as signaling analysis. Moreover, crosstalk between HUVECs and oral squamous cell carcinoma (OSCC) cells was investigated by indirect co-culture with condition medium or tumor cell derived exosomes. Our results showed that overexpression of RMND5A reduced the proliferation, migration and tube formation ability of HUVECs by inhibiting the activation of ERK and NF-κB pathway. Interestingly, OSCC cells can inhibit RMND5A expression of endothelial cells via exosomal miR-21. In summary, our present study unveils that OSCC cells can activate endothelial cells via exosomal miR-21/RMND5A pathway to promote angiogenesis, which may provide novel therapeutic targets for the treatment of OSCC.

**Supplementary Information:**

The online version contains supplementary material available at 10.1186/s12903-024-03852-3.

## Introduction

Ubiquitination is a complex process that involves the sequential actions of several enzymes [1]. First, ubiquitin is activated in an ATP-dependent process by an E1 ubiquitin-activating enzyme. Next, the activated ubiquitin is transferred to an E2 ubiquitin-conjugating enzyme. Finally, the E2 enzyme collaborates with an E3 ubiquitin ligase to transfer the ubiquitin molecule onto the target protein. The multi-subunit carboxy-terminal to LisH (CTLH) complex is a newly discovered E3 ligase, which is composed of at least Ran-binding protein M (RanBPM), muskelin, WD repeat-containing protein 26 (WDR26), armadillo repeat-containing protein 8 (ARMC8) α/β, GID4, required for meiotic nuclear division 5 A (RMND5A) and macrophage erythroblast attacher (MAEA) [2, 3]. The E3 ligase activity of the CTLH complex is dependent on RMND5A and MAEA. RMND5A harbors the LisH/CTLH motifs and contributes to microtubule dynamics, cell migration, nuclear motility and chromosome segregation. RMND5A has been known to be important for HeLa cell migration by interacting with RanBPM and stabilizing Exportin-5 protein [4]. Meanwhile, deletion of RMND5A promotes HEK293 cell proliferation via c-RAF/ERK signaling [5]. Also, RMND5A has been proven as a potential prognostic marker in breast cancer and pancreatic adenocarcinoma [6, 7]. However, the role of RMND5A in endothelial cells has not been reported.

Angiogenesis is the formative process of new blood vessels from the pre-existing vasculature [8]. Angiogenesis is a critical event in tumor progression by supplying oxygen and nutrients to tumor cells [9]. It is well known that endothelial cell (EC) proliferation and migration are required for angiogenesis [10]. Of interest, many studies have found that tumor cells can regulate angiogenesis via direct or indirect communication with endothelial cell [11, 12]. Based on the reported function of RMND5A, in this study, we investigated the role of RMND5A in endothelial cells and the underlying mechanisms. In addition, we found that oral squamous cell carcinoma (OSCC) cells could regulate RMND5A expression in endothelial cells via exosomal microRNA-21 (miR-21).

## Materials and methods

### Cell culture

As we previously described, human umbilical vein endothelial cells (HUVECs) were isolated from human umbilical cord veins [13]. This study has been approved by the review board of the ethics committee of the Hospital of Stomatology, Wuhan University, and the formed consent was signed by the parents of newborns. HUVECs were cultured in endothelial cell medium (ECM, Sciencell) containing 10% fetal bovine serum (FBS) and 1% endothelial cell growth supplement (ECGs). OSCC cell lines CAL27 and SCC25 were purchased from the American Tissue Culture Collection (ATCC). CAL27 cells were cultured in dulbecco’s modified eagle medium (DMEM) supplemented with 10% FBS. SCC25 cells were cultured in the mixture of DMEM and Ham’s F12 in equal proportion supplemented with 10% FBS. All the cells were grown in a humidified incubator at 37 °C and 5% CO2.

### Constructs and viral infection of HUVECs

Human RMND5A cDNA (NM_022780.4) was amplified by PCR and subcloned into a lentiviral pHS-AVC vector (pLV-hef1a-mNeongreen-P2A-Puro-WPRE-CMV-3xFlag) containing 3xFlag (Beijing Syngentech Co., Ltd.) (Beijing, China). 293FT cells and polyethylenimine (PEI) reagent was used for virus packaging. For RMND5A overexpression, HUVECs were infected with viral particles packaged with the recombinant plasmid, and 10 µg/ml polybrene (Sigma-Aldrich, Darmstadt, Germany) was used to enhance the efficiency of viral infection. After 48-h infection, the positively infected cells were selected with puromycin (InvivoGen, CA, USA).

### Transient miRNA transfection

Hsa-miR‑21‑5p mimics, hsa-miR‑21‑5p inhibitor and negative control (NC) were purchased from Guangzhou RiboBio Co., Ltd. (Guangzhou, China). HUVECs and OSCC cell lines CAL27 and SCC25 were transiently transfected with riboFECT^TM^CP Transfection Kit (RiboBio) at the concentration of 50 nM (miR-21 mimic and NC) or 100 nM (miR-21 inhibitor and NC).

### Real-time quantitative PCR (qPCR)

Total RNA of HUVECs was extracted using FastPure Cell/Tissue Total RNA Isolation Kit (Vazyme, Jiangsu, China). As we previously described [14], for mRNA detection, 1000 ng of RNA was reversely transcribed to cDNA by using the HiScript III RT SuperMix for qPCR (+ gDNA wiper) (Vazyme, Jiangsu, China). Obtained cDNA was amplified with Taq Pro Universal SYBR qPCR Master Mix (Vazyme, Jiangsu, China). GAPDH was selected as an internal control. MiRNA was synthesized into cDNA with specific RT primers and combined with specific forward primers for amplification by using Bulge-Loop™ miRNA qRT-PCR Starter Kit (RiboBio, Guangzhou, China). U6 was selected as an internal control. The primers of GAPDH, RMND5A, and RanBPM were synthesized by Tsingke Biotechnology Co., Ltd. U6 and miR-21 primers were synthesized by Guangzhou RiboBio Co., Ltd. Quant Studio 6 Flex Real-Time PCR System (Applied Biosystems, CA, USA) was used for real-time qPCR. All the primers’ sequences were shown in Table 1.

**Table 1:** Primer sequences used for real-time PCR

Gene	Forward (5’-3’)	Reverse (5’-3’)
RMND5A	TTTACACGGGATGCTTGTGC	ACTCCAGTACACTGCCTCTG
RanBPM	GCCCAGTTGGAAATCAGCTT	CTGATCGAGCCATCAGTCCT
GAPDH	GCTCTCTGCTCCTCCTGTTC	ACGACCAAATCCGTTGACTC

### Western blot analysis (WB)

According to our previous study [15], 20 µg of total protein was loaded for SDS-PAGE electrophoresis and transferred to the PVDF membrane at a voltage of 110 V (90 min). After that, the membrane was blocked in 5% skimmed milk for 1 h, and incubated with primary antibodies (anti Flag: #2064, Dia-An Biotechnology, 1:3000; anti-NF-κB: #8242, Cell Signaling Technology, 1:1000; anti-phos NF-κB: #3033, Cell Signaling Technology, 1:1000; anti-ERK: #4695, Cell Signaling Technology, 1:2000; anti-phos ERK: #4370, Cell Signaling Technology, 1:3000; anti-GAPDH: sc-365,062, Santa Cruz Biotechnology, 1:2000) at 4 ^o^C overnight, followed by incubation with HRP-conjugated secondary antibodies at room temperature for 1 h. The protein signals were detected with ECL kit. GAPDH was used as a loading control.

### MTT assay

HUVECs were seeded in a 96-well plate at 5 × 10^3^ cells per well and cultured in ECM plus 10% FBS and different doses of ECGs for 3 days. Subsequently, 20 µl of 3-(4,5- dimethylthiazole-2-yl)-2,5-diphenyl tetrazolium bromide solution (MTT) (5 mg/ml) was added to each well at 37 °C for 4 h away from light. After removing the supernatant, 150 µl of dimethyl sulfoxide (DMSO) was added to each well. The absorbance was measured on Bio-Tek microplate reader at 490 nm.

### Wound-healing assay

According to our previous study [16], HUVECs were seeded in 6-well plates and vertically scraped with a 200-ul sterile micropipette tip at 80-90% confluence. After that, the cells were gently rinsed with PBS and cultured with serum-free ECM media at 37 °C and 5% CO_2_. At 0 and 24 h, the cells were photographed under a phase microscope and the migrated cells were counted.

### In vitro tube formation assay

According to our previous study [16], the cool 96-well plate was coated with 50 ul of Matrigel (BD Biosciences) and placed in a 37 °C incubator for 45 min. Next, HUVECs were seeded in triplicate for 10 h and capillary-like structures were quantified under a phase microscope (Olympus).

### Isolation and size-measurement of exosomes

CAL27 and SCC25 cells were cultured in exosome-depleted serum. The collected media was centrifuged at 2,000 g for 10 min, and the supernatant was centrifuged at 16,000 g for 1 h at 4 °C to remove the cell debris and big vesicles. After that, the supernatant was ultracentrifuged at 100,000 g for 2 h at 4 °C. The obtained pallet was suspended in PBS and stocked at -80 °C. Meanwhile, a little bit of pallet solution was diluted and loaded into a NanoSight NS300 to measure the particle size.

### Statistical analysis

All data are presented as mean ± SD and analyzed by using GraphPad Prism 9 (San Diego, CA, USA). Student’s *t*-test was used when comparing two groups, while one-way ANOVA followed by post hoc tests was used if more than two groups. *P* < 0.05 was considered statistically significant.

## Result

### RMND5A inhibits the proliferation, migration and tube formation ability of HUVECs

**Fig. 1:**
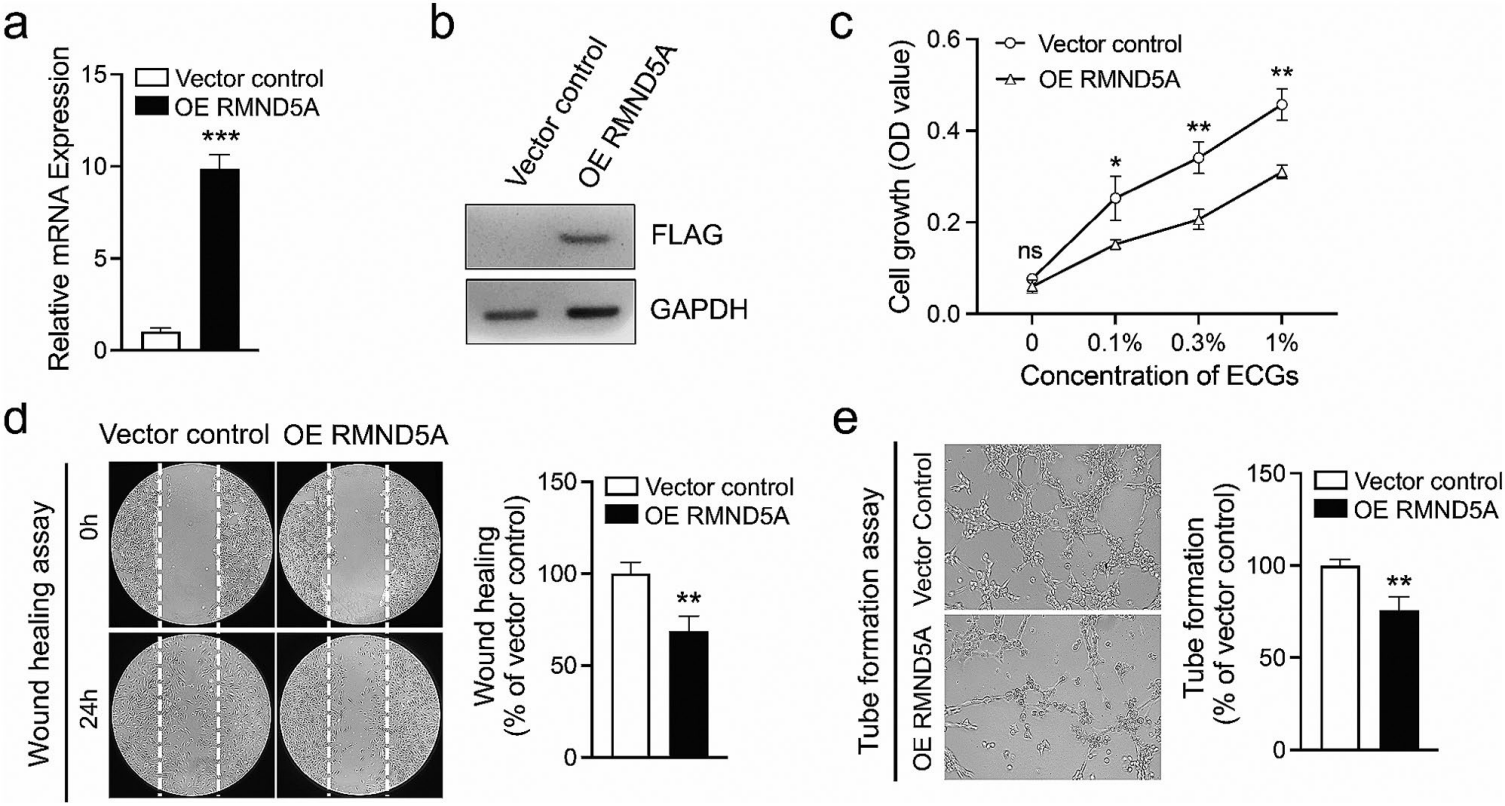
RMND5A inhibits the proliferation, migration and tube formation of HUVECs. (**a**, **b**) Overexpression of Flag-tag RMND5A in HUVECs via lentiviral infection. Real-time PCR (**a**) and WB (**b**) data proves the elevated expression of RMND5A in HUVECs. (**c**) MTT assay shows ECGs dependent growth of HUVECs. By comparison with vector control group, overexpression of RMND5A significantly inhibits the proliferation of HUVECs under the condition of ECGs. (**d**) Would healing assay shows that overexpression of RMND5A significantly inhibited the migration ability of HUVECs. (**e**) Overexpression of RMND5A reduces the tube formation ability of HUVECs evidenced by decreased capillary-like structures. Data is expressed as mean ± SD. ns, not significant; *P < 0.05; **P < 0.01; ***P < 0.001

To investigate the role of RMND5A in endothelial cells, we overexpressed Flag-tag RMND5A in HUVECs via lentiviral infection. Real-time PCR and WB data proved the elevated expression of RMND5A in HUVECs (Fig. 1a and b). Our MTT data showed ECGs dependent growth of HUVECs (Fig. 1c). ECGs induced concentration dependent proliferation of HUVECs under a concentration of 1% (Fig. 1c). By comparison with vector control group, overexpression of RMND5A significantly inhibited the proliferation of HUVECs under the condition of ECGs (Fig. 1c). Would healing assay showed that overexpression of RMND5A significantly inhibited the migration ability of HUVECs (Fig. 1d). In addition, overexpression of RMND5A reduced the tube formation ability of HUVECs evidenced by decreased capillary-like structures (Fig. 1e).

### RMND5A inhibits the activation of ERK and NF-κB in HUVECs

**Fig. 2:**
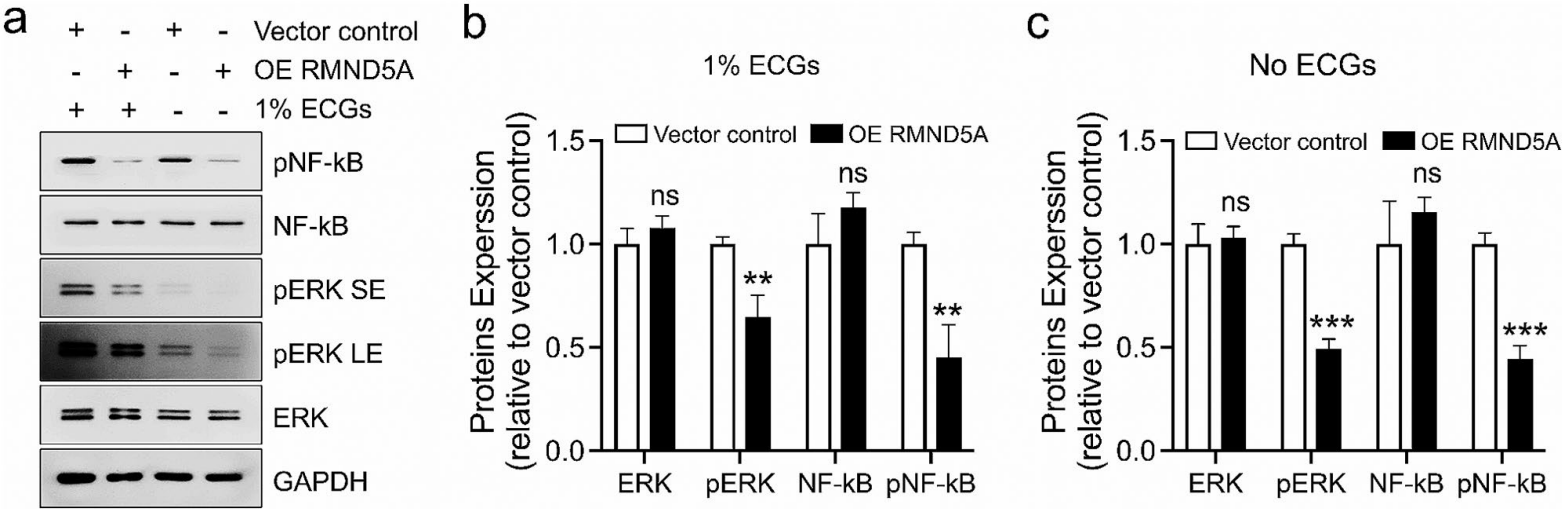
RMND5A inhibits the activation of ERK and NF-κB in HUVECs. WB data shows that RMND5A overexpression in HUVECs significantly decreases both ERK and NF-κB phosphorylation levels under the condition of ECM media with 1% ECGs (**a**, **b**) or without ECGs (**a**, **c**). Data is expressed as mean ± SD. ns, not significant; **P < 0.01; ***P < 0.001

Numerous studies have demonstrated the critical role of ERK and NF-κB pathways in the regulation of cell proliferation and migration [17, 18]. Recently, it was reported that RMND5A can regulate ERK activation via c-RAF ubiquitination in HeLa cells [5]. To explore the mechanism on the effects of RMND5A in HUVECs, we compared the activation of ERK and NF-κB. Our WB data showed that the phosphorylation levels of both ERK and NF-κB were significantly decreased in the HUVECs overexpressing RMND5A compared with vector control cells in the ECM media with 1% ECGs (*P* < 0.01) (Fig. 2a and b) or without ECGs (*P* < 0.001) (Fig. 2a and c) for 4 h at 37 ^o^C. These results revealed that RMND5A may inhibit endothelial cell proliferation, migration and tube formation by regulating ERK and NF-κB pathways.

### OSCC cell-derived exosomes inhibit RMND5A gene expression of HUVECs

**Fig. 3:**
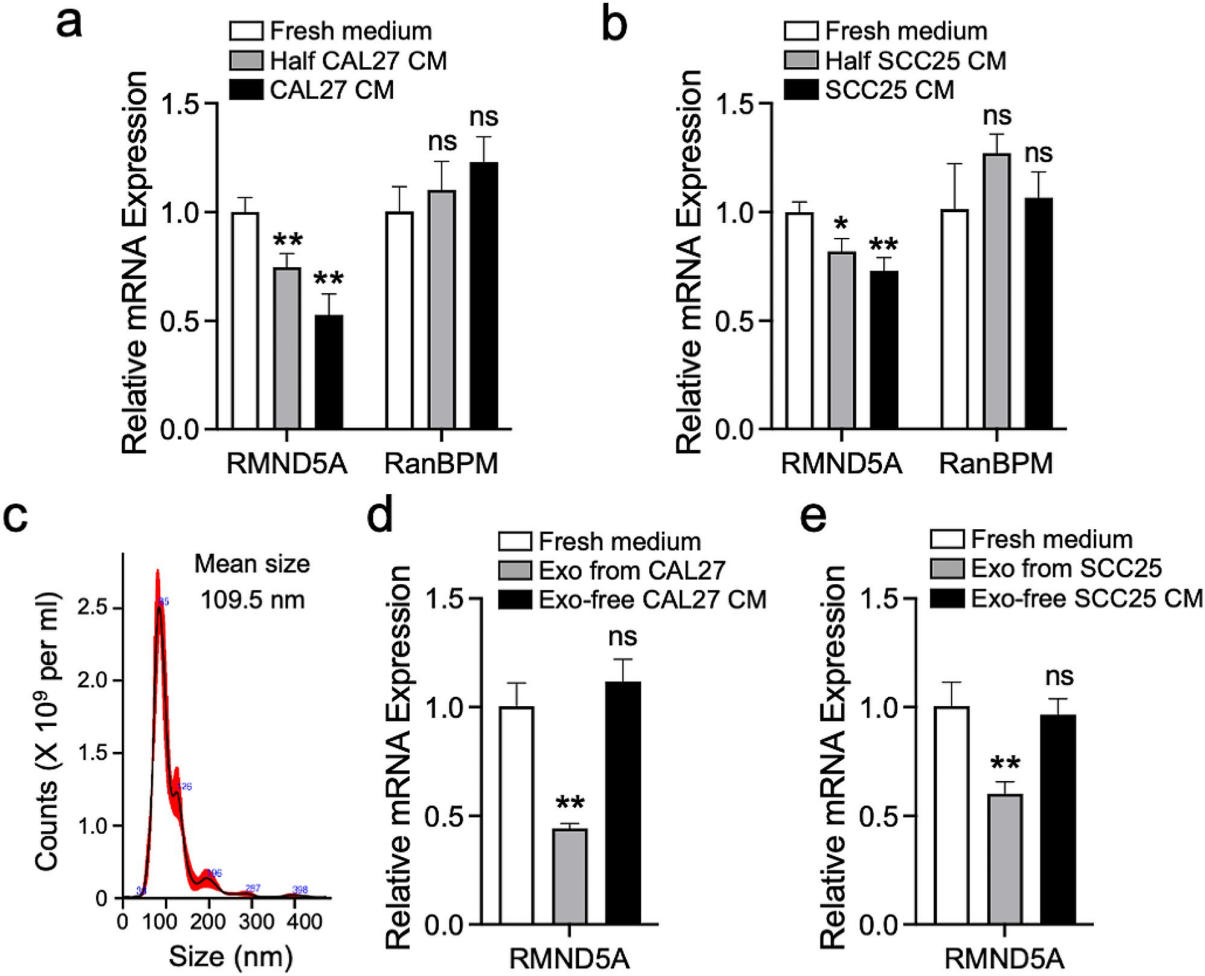
OSCC cell-derived exosomes inhibit RMND5A gene expression of HUVECs. (**a**, **b**) CAL27 (**a**) and SCC25 (**b**) cell culture supernatant (condition media, CM) dose-dependently decreases RMND5A gene expression in HUVECs after 24-h treatment, but does not change RanBPM gene expression. (**c**) The exosomes are purified from the condition media by differential centrifugation and confirmed by Nanosight. (**d**, **e**) The exosomes from CAL27 (**d**) and SCC25 (**e**) cells, but not exosome-depleted CM, significantly decrease RMND5A gene expression in HUVECs after 24-h treatment. Data is expressed as mean ± SD. ns, not significant; *P < 0.05; **P < 0.01

Crosstalk between tumor cells and endothelial cells is important to tumor development [19, 20]. Based on above results, we further investigated the effects of OSCC cells on RMND5A expression in HUVECs. Interestingly, the cell culture supernatant (condition media, CM) from either CAL27 or SCC25 cells dose-dependently decreased RMND5A gene expression in HUVECs after 24-h treatment. However, the gene expression of RanBPM, another subunit of CTLH complex, was not changed (Fig. 3a and b). Previous studies have reported that tumor-derived exosomes can target endothelial cells to promote angiogenesis [21, 22]. Therefore, we isolated the exosomes from the condition media by differential centrifugation and confirmed the size by Nanosight (Fig. 3c). Our data showed that the exosomes from either CAL27 or SCC25 cells significantly decreased RMND5A gene expression in HUVECs after 24-h treatment, while the exosomes depleted CM from OSCC cells cannot inhibit RMND5A expression (Fig. 3d and e). These results demonstrated that the inhibitory effect of OSCC cells on RMND5A expression of endothelial cells was exosome dependent.

### OSCC cell-derived exosomal miR-21 inhibits RMND5A gene expression of HUVECs

**Fig. 4:**
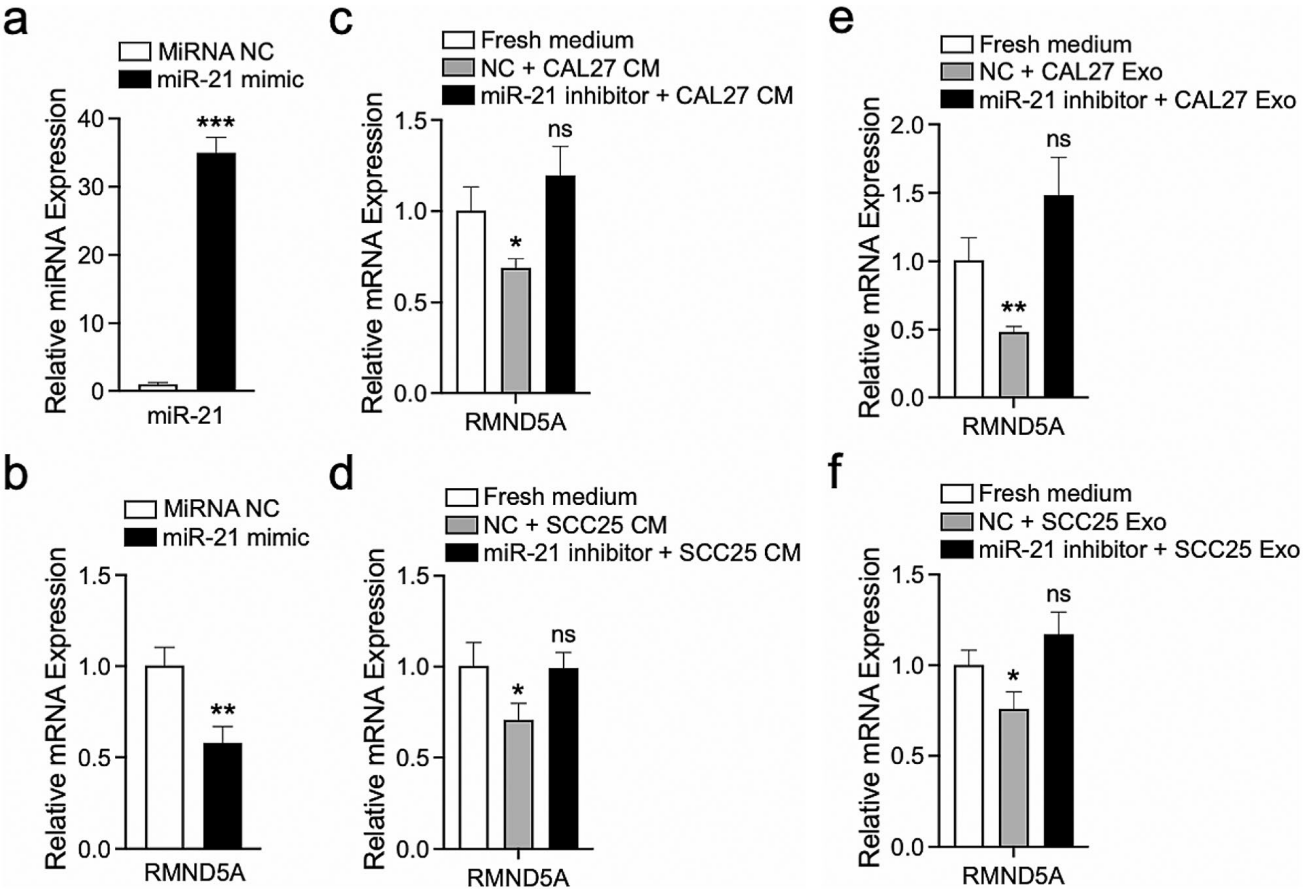
OSCC cell-derived exosomal miR-21 inhibits RMND5A gene expression of HUVECs. (**a**) Real-time PCR proves that miR-21 expression in HUVECs is upregulated after transfection with miR-21 mimic. (**b**) RMND5A gene expression is significantly decreased after overexpression of miR-21 using miR-21 mimic in HUVECs. (**c**, **d**) The condition media of miR-21 inhibitor transfected CAL27 (**c**) and SCC25 (**d**) cells cannot inhibit RMND5A expression of HUVECs. (**e**, **f**) The exosomes derived from miR-21 inhibitor transfected CAL27 (**e**) and SCC25 (**f**) had no significant effects on RMND5A gene expression of HUVECs. Data is expressed as mean ± SD. ns, not significant; *P < 0.05; **P < 0.01; ***P < 0.001

Exosomal miRNAs have been proven to play a critical role in tumor microenvironment by inhibiting gene expression [23]. Previous studies demonstrated that miR-21 is consistently overexpressed in a variety of cancers including OSCC [24], and RMND5A is a strong candidate target of miR-21 [25]. Meanwhile, miR-21 is found to be highly expressed in the CAL27- and SCC25-derived exosomes [26]. Our data showed that RMND5A gene expression was significantly decreased after overexpression of miR-21 using miR-21 mimic in HUVECs (Fig. 4a and b). Importantly, the condition media of miR-21 inhibitor transfected CAL27 and SCC25 cannot inhibit RMND5A expression of HUVECs (Fig. 4c and d). Consistently, the exosomes derived from miR-21 inhibitor transfected CAL27 and SCC25 cells had no significant effects on RMND5A gene expression of HUVECs (Fig. 4e and f). These data revealed that OSCC cells can inhibit RMND5A expression of endothelial cells via exosomal miR-21.

Furthermore, we found that CAL27-derived exosomes significantly enhanced the proliferation, migration and tube formation as well as ERK activation in HUVECs (Supplemental Fig. 1). More importantly, we noted a substantial decrease in these effects after silencing miR-21 in CAL27 cells, suggesting that miR-21 plays a critical role in modulating the pro-angiogenic properties of OSCC-derived exosomes.

## Discussion

Previous studies have demonstrated that RMND5A expression is significantly higher in the tumor tissues of pancreatic adenocarcinoma, stomach adenocarcinoma and thymoma compared to normal tissues, respectively [7]. In addition, RMND5A is reported to positively regulate the migration of HeLa and pancreatic adenocarcinoma cells [4, 7]. However, the function of RMND5A in endothelial cells has not been reported. In this study, we found that reversely, overexpression of RMND5A inhibited the proliferation, migration and tube formation of endothelial cells. Mechanistically, our results showed that overexpression of RMND5A in endothelial cells attenuated ERK and NF-κB activities, which are classical positive regulators in the cell cycle, motility and tube formation of endothelial cells [27–29]. These data on the signaling pathway is very consistent with a recent study that RMND5A is an E3 ligase of c-RAF to regulate c-RAF degradation thereby ERK activation [5].

Endothelial cell is a key player in angiogenesis [30]. Under normal conditions, most of endothelial cells and vascular system remain quiescent [31]. However, in tumor microenvironment, tumor cells can activate normal quiescent endothelial cells for angiogenesis in a paracrine manner such as cytokine, growth factor and extracellular vesicle (EV) [32]. Exosomes, a small size subset of EVs around 30 ~ 150 nm in diameter, can transport proteins and nucleic acids including miRNAs as mediators in cell-to-cell communication [33]. So far, several exosomal miRNAs have been identified to influence angiogenesis and extracellular matrix remodeling in the tumor microenvironment via multiple signaling pathways. OSCC derived exosomal miR-221 can target phosphoinositide-3-kinase regulatory subunit 1 to enhance tube formation ability of HUVECs [34]. Meanwhile, another study reveals that OSCC derived exosomes can promote tumor angiogenesis by transfer miR-210-3p and targeting PI3K/AKT pathway [35]. Besides, OSCC derived exosomes are rich in miR-21, which displays oncogenic activity and acts as an angiogenesis inducer via activating AKT and ERK signaling pathway [36]. In this study, we proved that miR-21 can downregulate RMND5A expression in endothelial cells as predicated in previous study [37]. Moreover, we found that OSCC cells can regulate RMND5A expression in endothelial cells by secreting exosomal miR-21. For the function assay, silencing miR-21 in OSCC cells led to a marked reduction in the pro-angiogenic activities of OSCC derived exosomes. The residual pro-angiogenic effects of OSCC derived exosomes may be attributed to other enriched miRNAs.

There are still some limitations in this study. For example, RMND5A KO mice could be generated to investigate the effects of RMND5A on vascular system in vivo. Furthermore, we are generating a specific and sensitive antibody against RMND5A to explore the role of miR-21/RMND5A in OSCC progression and elucidate the clinical significances of RMND5A expression, such as the correlation among endothelial RMND5A expression with microvessel density in OSCC tissues and prognosis of OSCC patients.

In summary, our present study is the first to report inhibitory effects of RMND5A on the proliferation, migration and tube formation of endothelial cells via inhibiting ERK and NF-κB activation. Moreover, OSCC cells can activate endothelial cells via exosomal miR-21/RMND5A pathway to promote angiogenesis. Our present study unveils new mechanisms of tumor angiogenesis and may provide novel therapeutic targets for the treatment of OSCC.

### Electronic supplementary material

Below is the link to the electronic supplementary material.

**Supplementary Material 1: Supplemental Figure 1.** OSCC-derived exosomes significantly enhance the proliferation of HUVECs, migration and tube formation as well as ERK activation in HUVECs, while these effects are reduced after silencing miR-21 in OSCC cells. (a) MTT assay. (b) WB assay. (c, d) Would healing assay. (e, f) Tube formation assay. Data is expressed as mean ± SD. ns, not significant; *, P < 0.05; **, P < 0.01

Supplementary Material 2

## Data Availability

The datasets used and/or analyzed during the current study are available from the corresponding author on reasonable request.
