# Supplementary material for: Oral cancer cell to endothelial cell communication via exosomal miR-21/RMND5A pathway
Source: BMC Oral Health. 2024 Jan 16;24:82. doi: 10.1186/s12903-024-03852-3 (PMC10790467; doi:10.1186/s12903-024-03852-3)
Supplement: Supplementary file 1 — Supplementary Material 1: Supplemental Figure 1. OSCC-derived exosomes significantly enhance the proliferation of HUVECs, migration and tube formation as well as ERK activation in HUVECs, while these effects are reduced after silencing miR-21 in OSCC cells. (a) MTT assay. (b) WB assay. (c, d) Would healing assay. (e, f) Tube formation assay. Data is expressed as mean ± SD. ns, not significant; *, P < 0.05; **, P < 0.01 [file 12903_2024_3852_MOESM1_ESM.docx]

**Oral cancer cell to endothelial cell communication via exosomal miR-21/RMND5A pathway**

Yu-qi Sun^a,1^, Bing Wang^a,1^, Lin-wei Zheng^a^, Ji-hong Zhao^a,b,^*, Jian-gang Ren^a,b,^*

^a^ The State Key Laboratory Breeding Base of Basic Science of Stomatology (Hubei-MOST) & Key Laboratory of Oral Biomedicine Ministry of Education, School & Hospital of Stomatology, Wuhan University, Wuhan, China, 430079

^b^ Department of Oral and Maxillofacial Surgery, School & Hospital of Stomatology, Wuhan University, Wuhan, China, 430079

^1^Authors contributing equally to this article.

*Correspondence: Ji-hong Zhao and Jian-gang Ren, School & Hospital of Stomatology, Wuhan University, No. 237 Luoyu Road, Wuhan 430079, China. Tel: 86-27-87686386; Fax:86-27-87873260. Email: jhzhao988@whu.edu.cn; rjg19870708@whu.edu.cn.

Conflict of Interest: None

Grant numbers and sources of support: This work was funded by the grants from National Natural Science Foundation of China to Dr. J.G. Ren (81600385) and Prof. J.H. Zhao (81671008).


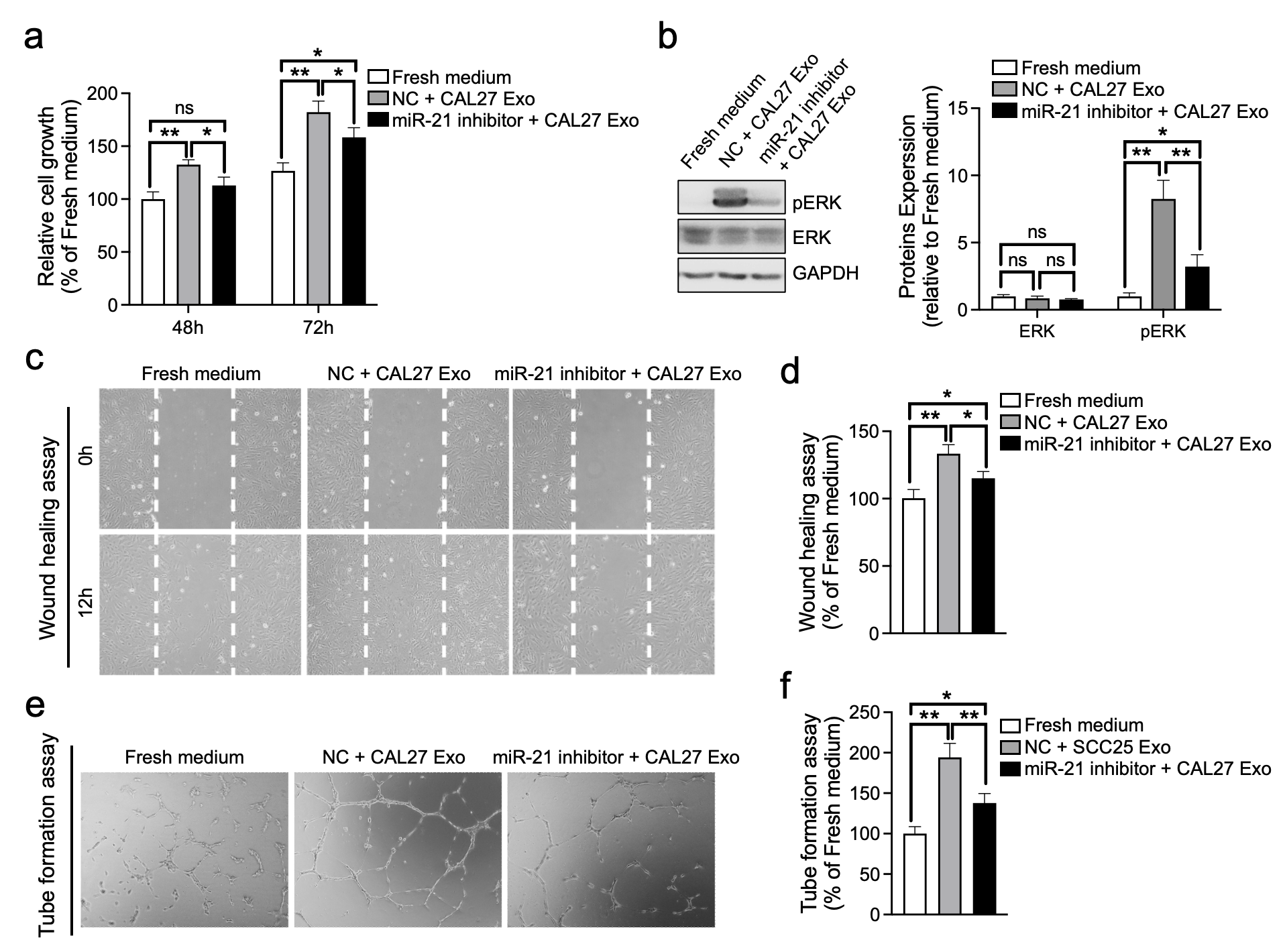


Supplemental Figure 1. OSCC-derived exosomes significantly enhance the proliferation of HUVECs, migration and tube formation as well as ERK activation in HUVECs, while these effects are reduced after silencing miR-21 in OSCC cells. (a) MTT assay. (b) WB assay. (c, d) Would healing assay. (e, f) Tube formation assay. Data is expressed as mean ± SD. ns, not significant; *, *P* < 0.05; **, *P* < 0.01.
