# Supplementary material for: Oral cancer cell to endothelial cell communication via exosomal miR-21/RMND5A pathway
Source: BMC Oral Health. 2024 Jan 16;24:82. doi: 10.1186/s12903-024-03852-3 (PMC10790467; doi:10.1186/s12903-024-03852-3)
Supplement: Supplementary file 2 — Supplementary Material 2 [file 12903_2024_3852_MOESM2_ESM.pdf]

Fig. 1b

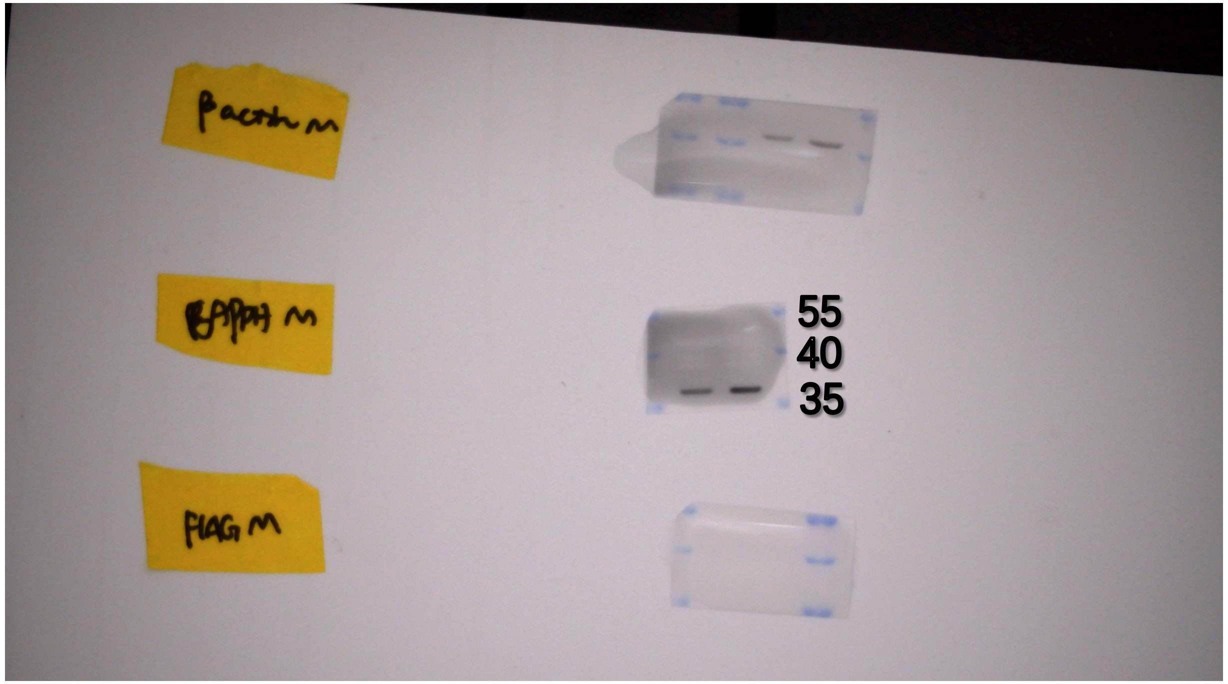

GAPDH

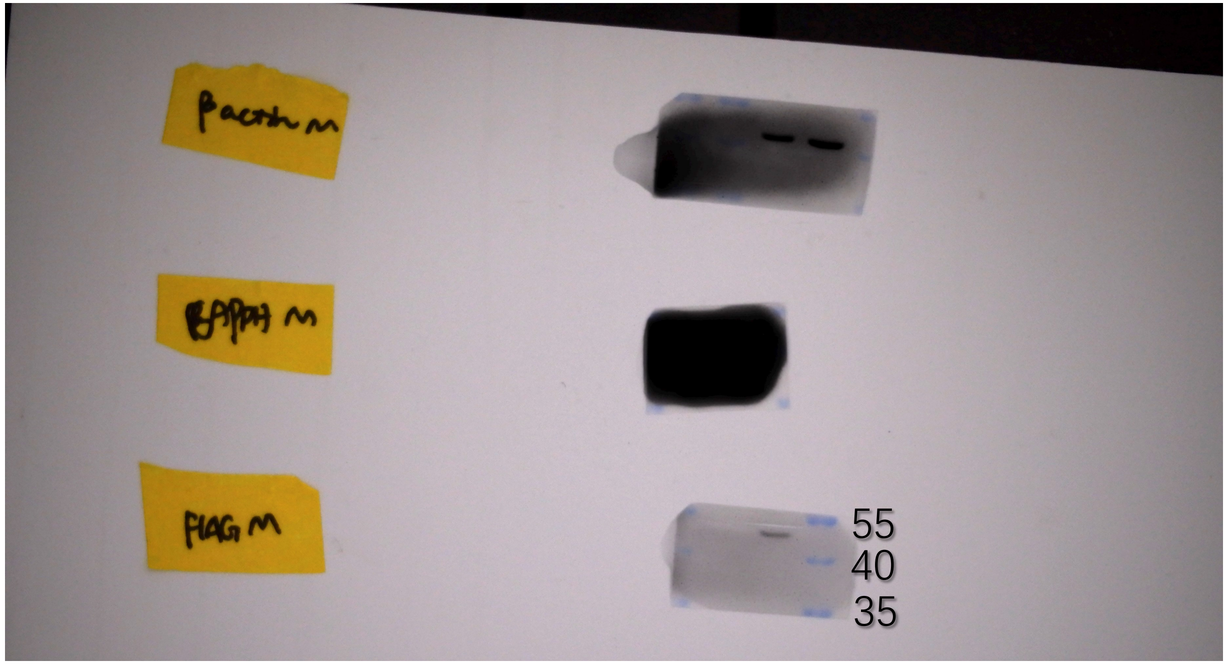

FLAG

Fig.2a

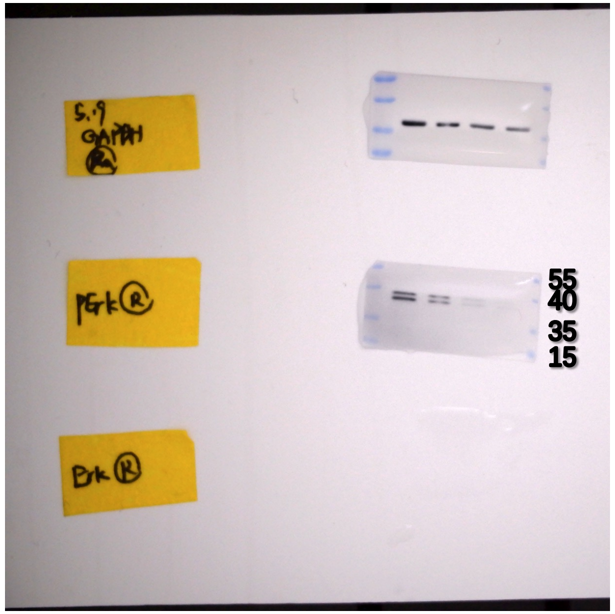

pERK

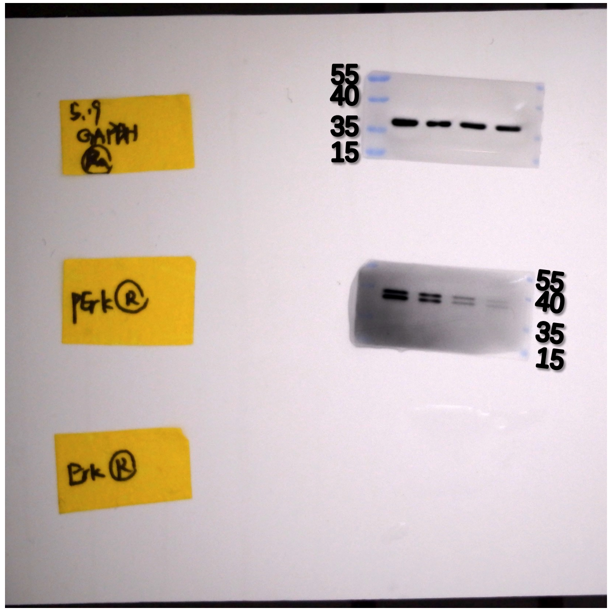

GAPDH

pERK

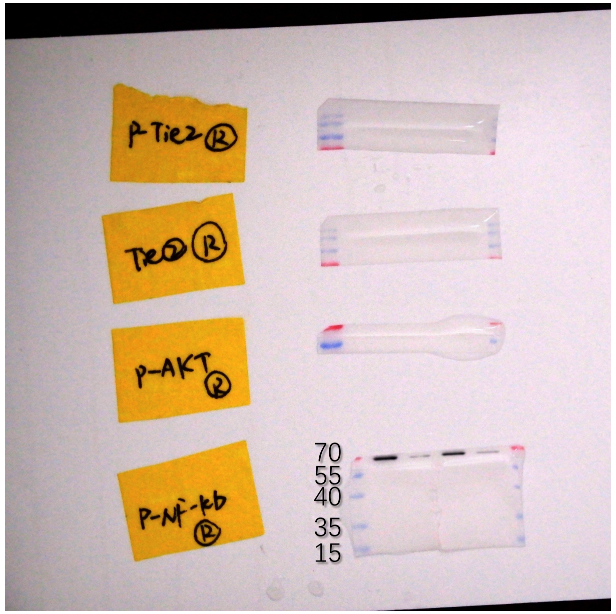

pNF-KB

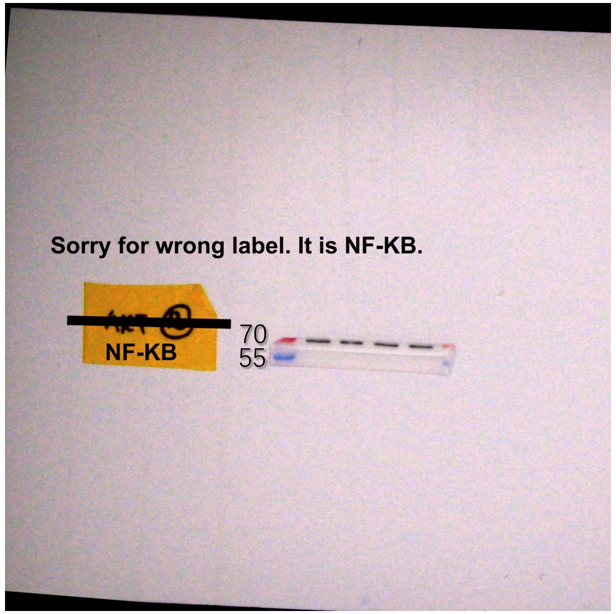

NF-KB

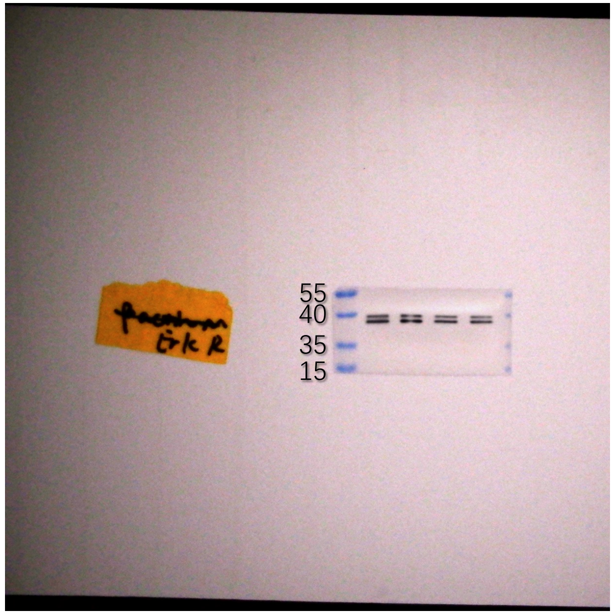

ERK

Supplemental Fig.1b

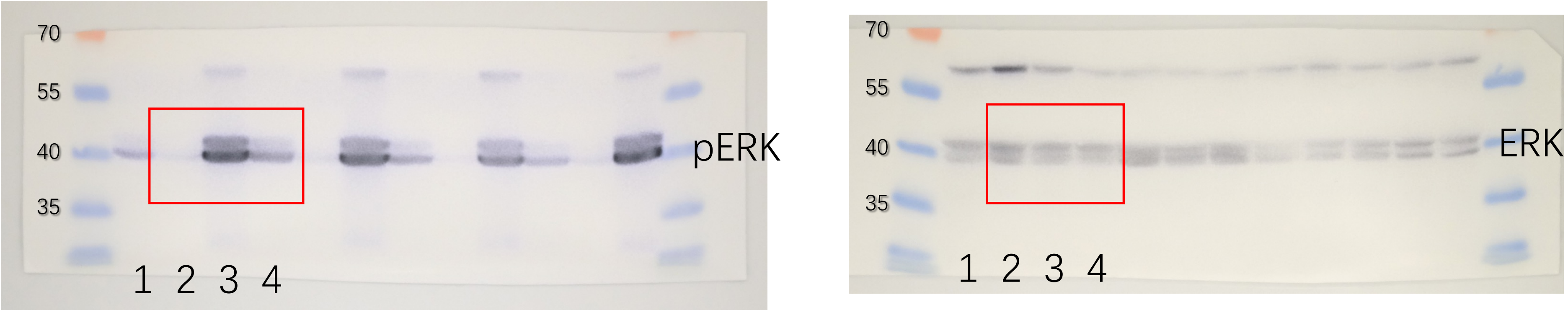

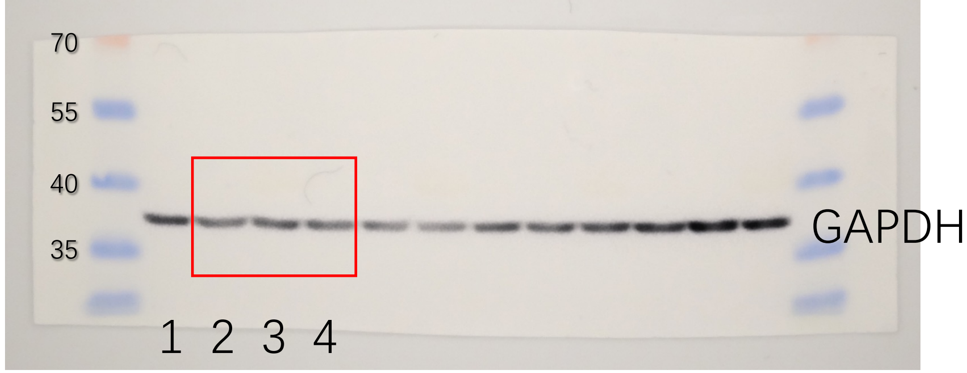

1. VEGF treated
2. Fresh medium
3. NC + SCC25 Exo
4. miR-21 inhibitor + SCC25 Exo
